# The importance of Open Access publishing in the field of Linguistics for spreading scholarly knowledge and preserving languages diversity in the era of the economic financial crisis

**DOI:** 10.3389/fnbeh.2013.00091

**Published:** 2013-08-02

**Authors:** Nicola L. Bragazzi

**Affiliations:** ^1^Department of Neuroscience, Rehabilitation, Ophthalmology, Genetics, Maternal and Child Health (DINOGMI), Section of Psychiatry and School of Public Health, Department of Health Sciences (DISSAL), University of GenoaGenoa, Italy

I read with great interest the article recently published on “Frontiers in Behavioral Neuroscience,” written by Haspelmath and concerning the Open Access (OA) with a particular emphasis from the field of theoretical linguistics (Haspelmath, 2013). However, there are some points in which I disagree with him and I would like to discuss these points as well as I would like to put forth further arguments, adding also an Italian perspective to the topic.

First, the author claims that OA has been marginally noticed in the field of theoretical linguistics, at least in his university context. Moreover, he describes the reactions of “disbelief and indignation” of his colleagues when they hear that they should pay a fee for having their manuscripts published. However, I maintain that this picture for OA journals (OAJs) devoted to linguistics is not exactly true: if we search for linguistics-related OAJs on the Directory of OAJs (DOAJ) database, the largest database collecting OAJs, we discover that only 11 journals have a publishing fee (out of 213, that is to say approximately the 5.2%), while for 4 of them there are no available information (about the 1.9%).

OA seems to be a vivid and expanding reality, and in the field of linguistics it is highly supported by initiatives such as the OA Initiative in Linguistics (OALI), coordinated by the German linguist Stephen Müller (Müller, 2012) and by the same Haspelmath.

Also in Italy, as in Germany, toll-based or subscription-based publishing has revealed its inefficiency: costs that have inflated and increased out of proportion (Giunta, 2010). This kind of publishing is no longer sustainable and affordable. OA seems more cost-effective.

Another point I would like to put forward is that OA could provide an exceptional opportunity and a platform for preserving languages diversity. Article Processing Charges (APCs) for OAJs are (completely or partially) waived for researchers coming from underdeveloped or developing countries, even if as we have already seen that are no publishing fees for the majority of linguistics-related OAJs. OA is growing especially in underdeveloped countries and developing nations (Bayry, 2013), since for them it represents a great opportunity to make their voices heard. The service offered to scholars in these countries is undoubtedly of value: they can freely access scientific content and distribute and communicate with other scholars. Researchers from emerging countries could exploit the benefits of OA for sharing information about languages at risk of extinction in a cost-effective way, uploading also audio-resources and creating a great digital archive. UNESCO has already recognized the importance of the Internet and of other new media to fill the linguistic divide, to preserve multiculturalism and to foster “pluralistic, equitable, open and inclusive knowledge societies” (UNESCO).

In conclusion, the importance of OA in the era of a severe financial and economic crisis is acknowledged. The current crisis is imposing cuts of funding, recruitment/hiring and turnover freezes, and some countries such as Greece have lost access to prestigious journals (Trachana, 2013). OA can overcome the hurdles and the barriers to the spread and the dissemination of the knowledge. OA could provide an excellent platform for delivering and sharing scholarly data and results, contributing at the same time to make the cyberspace a more multilingual reality.
